# Efficiency of two constructs called "fear of disease" and "perceived severity of disease" on the prevention of gastric cancer: Application of protection motivation theory

**Published:** 2015

**Authors:** Mohamad Hosein Baghiani- Moghadam, Seyed Jalil Seyedi-Andi, Javad Shokri-Shirvani, Sorayya Khafri, Reza Ghadimi, Hadi Parsian

**Affiliations:** 1 Department of Health Education and Promotion, Faculty of Health, Shahid Sadoughi Yazd University of Medical Sciences, Yazd, Iran.; 2 Department of Internal Medicine, Faculty of Medicine, Babol University of Medical Sciences, Babol, Iran.; 3 Department of Statistics, Faculty of Medicine, Babol University of Medical Sciences, Babol, Iran.; 4 Social Determinants of Health Research Center, Health Research Institute, Babol University of Medical Sciences Babol, Iran.; 5 Department of Biochemistry, Faculty of Medicine, Babol University of Medical Sciences, Babol, Iran.

**Keywords:** Perceived Severity, Fear, Gastric Cancer, Protection Motivation Theory

## Abstract

**Background:**

Among all cancers, malignancies of gastrointestinal tract are the most common cancer among Iranian population. Dietary behavior is thought to be the most important risk factor in gastric cancer. Fear and perceived severity are two important constructs of the protection motivation theory (PMT). Despite the evidence of the impact of these two constructs in modifying dietary habits against gastric cancer, their efficiency is not well established. Therefore, the present study was designed to determine the efficiency of the mentioned constructs.

**Methods:**

This cross-sectional study was performed on 360 participants (180 males and 180 females) aged over 30 years old who presented to health centers in Babol, Iran in 2014. They were selected by a cluster sampling method in a population covered by health centers in Babol. Data collection was done using a questionnaire with acceptable reliability and validity, designed by a researcher based on two constructs of protection motivation theory. The data were analyzed by SPSS Version 20 using descriptive and analytical statistics such as ANOVA, linear and logistic regression analysis.

**Results:**

The participants who entered in the study achieved 38.6 and 69.7% of the scores of fear and perceived severity, respectively. There was a significant difference between perceived severity with level of education (p<0.05). There was a significant inverse correlation between perceived severity with nutritional high risk behavior associated with gastric cancer in the significant level of 0.05 (r=-0.165). The constructs of perceived severity and fear predicted 38% of the variance of nutritional high risk behaviors associated with gastric cancer.

**Conclusion:**

Constructs of fear and perceived severity of protection motivation theory with predicting 38% of the variance of nutritional high risk behaviors had an effective role against gastric cancer and may help in the design and implementation of educational programs for the prevention of gastric cancer.

Among all cancers, malignant conditions of gastrointestinal tract are the most common among Iranian population. Dietary behavior is the most important risk factor predisposing to gastric cancer. The risk of gastric cancer increases by eating more salt foods, salt and processed meats and reducing consumption of fruits and vegetables. In addition, there is an inverse association between consumption of green tea, vitamin C and gastric cancer (1). One of the most common causes of death in the age group above 50 years old is cancer where lifestyle changes to prevent it (2).

Annually, 1.6 million new cases of cancer are diagnosed worldwide and 1.3 million of them die of this cancer (3). Gastric cancer after lung, breast and colorectal cancer is the fourth most common cancer globally and it is the second leading cause of death (4). Gastric cancer causes "two-thirds of cancer deaths and they occur in less developed countries (5). 50% of our common cancers are in the digestive tract and gastric cancer is the most common one (6-8). Proper diet and food preparation methods may reduce the incidence of gastric cancer (9), where behavioral and nutritional risk factors discussed about it (10). Diet is the most important environmental factor in gastric cancer (11). The use of smoked foods, salt-plated and pickled foods (12, 13) with high fat and low in fiber, are the risk factors of gastric cancer and have increased. Nutrition is the cause of 30 to 40 percent incidence of cancers (14). Nutritional education is an important element in health promotion and disease prevention programs (15). 

Increasing the effectiveness of health education depends on the proper use of theories and models (16). For research and planning of a study about modifying behavior elements, it is essential to use a reliable and popular model (17). One of the most important theories and models used in the study of social cognitive and motivational factors influencing the behavior of individuals is protection motivation theory (PMT) which was described by Rogers in 1975 to explain the effects of fear on health on attitudes and behavior. This theory includes two important steps, 1: threat appraisal (perceived susceptibility, perceived severity, reward and fear) and 2: coping appraisal (self-efficacy, perceived response efficacy and response-cost) (18). Given that the most important issue in patients with gastric cancer is preventive behaviors, this theory is a comprehensive model to predict such behaviors. 

The basic principles of this theory are protective and preventive behaviors (19). Perceived severity is a person's belief that the threat is serious (20). In perceived severity, researchers measure the perceived negative consequences of high risk behavior (21). For example, a person might believe that smoking causes lung cancer and after a few years, death is certain. Fear is one mediator between perceived susceptibility, perceived severity and protected motivation. So understanding that this is vulnerable to a serious health threat, raised the high level of fear consequently and the motivation to perform preventive behavior increases (22). The data of Baghianimoghadam assessed the factors relevant to skin cancer. Preventive behavior in female high school students in Yazd based on protection motivation theory in 2010 showed a significant positive correlation of 0.01 between fear and perceived severity with protective behavior of skin cancer (23). Despite the high prevalence of gastric cancer in northern Iran and various socio-economic consequences for the individual, family and community, there was no study on the prevention of gastric cancer based on protection motivation theory (figure 1). Therefore, the present study aimed to explain the constructs of fear and perceived severity in the field of nutritional high risk behaviors associated to gastric cancer. 

**Fig 1:**
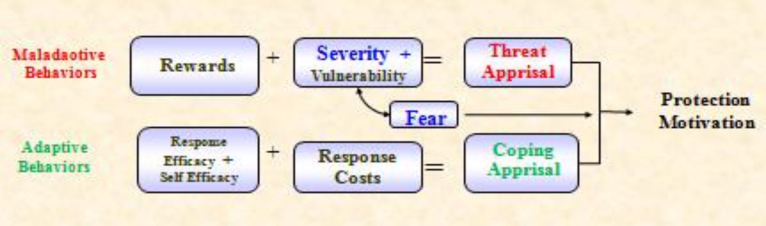
Protection Motivation Theory

## Methods

This cross-sectional study was performed on 360 participants (180 males and 180 females) age more than 30 years old who presented to health centers in Babol, Iran in 2014. The sample size was based on a statistical formula, according to 95% confidence level and 90% test power and the results of Brian T (24) of 60 individuals from each of the 6 selected medical centers of Babol and in total, 360 individuals were determined. The samples were selected via a multi-stage cluster sampling. Thus, the first step was to make a list of city health centers from urban health centers. Then randomly 6 health centers in various locations were selected. The next stage of every health center, 60 individuals (30 males and 30 females) were randomly selected through their health records. The inclusion criteria for all participants were: health services being covered in health centers in Babol, older than 30 years, no risk of serious diseases. Data collection was a researcher questionnaire with acceptable reliability and validity, designed by a researcher after a qualitative need assessment approach based on the protection motivation theory. The first part of the questionnaire was (questions 1 to 9) on demographic characteristics. The second part of the questionnaire was based on protection motivation theory, that included perceived severity (6 questions), fear (6 questions), and behavior (11 questions). Content validity was confirmed by the panel of experts and internal consistency of structures was provided based on a pilot study with 30 samples in which Cronbach’s alpha was greater than 0.7. There is a direct link between fear mediator and perceived severity. The included questions about fear was" I am worried that I may develop gastric cancer like several of my town mates and the question about the perceived severity was" Gastric cancer in the area where I live, is more common and serious ". The answers were rated on a Likert scale (0-4). Never, rarely (or seldom), sometimes (or usually, always) for fear construct - Totally agree, disagree, without comment, agree, totally agree for perceived severity construct) that the range of scores for these two constructs was 0-24. High risk behavior for gastric cancer was measured with 11 questions in which three questions in this regard were: 1- Do you eat fast food? 2- Do you eat pickled - foods? 3- Do you eat fried foods? To answer these questions, 4 answers were included: No (disagree), slight yes (slightly agree), moderate yes (moderately agree), strong yes (strongly agree). The scores for these questions were 0-3, with range of 0-33. The data were coded by computer and analyzed by SPSS 20 software using descriptive and analytic statistics such as ANOVA, linear and logistic regression analysis and a level of 0.05 was considered significant. The variables were entered into the regression model, P<0.01 and/or were considered clinically important (P<0.05).

## Results

According to the results of the current study of the participants who referred to health care centers of Babol, Iran, age group 31-40 years (66.1%) had the highest frequency age group over 60 years (2.8%) the least frequency. The lowermost percentage (5.3%) of the frequencies was applied to those clients who can read and write only. In this study, the mean score of fear, from the range of 0-24 scores, was 9.26±4.63, which shows that the cases of the study gained 38.58% of the possible scores. As well as the mean score of the perceived severity was 16.73±2.95, ranging from a score of 0-24 and indicate that it was accessible to 69.70% of the subjects. Table 1 represents the distribution of absolute and relative frequencies of the participants' answers to the questions about fear. Based on these results, it can be observed that "the concern of the possibility of the risk of getting gastric cancer like a number of his townmates (32.50%) " as well as "the fear and anxiety of the probability of the risk of gastric cancer due to poor nutritional status of residence (32.50%)" were reported topics related to fear of the subjects. The lowermost level of the perceived severity that the participants expressed to prevent the risk of gastric cancer was the "occurrence of the intense abdominal pains in terms of the incidence of the gastric cancer (61.75%)".

**Table 1:** Absolute and relative frequency distribution of the nutritional high risk behavior-based questions of fear on the risk of gastric cancer in patients over 30 years that referred to urban health care centers, Babol, Iran

**Options** **Questions of fear**	**Never**	**Sometimes**	**Some deal**	**Often**	**Always**	**Mean score**
I am often worried about being at the risk of gastric cancer like my several of town mates	109 (30.3)	88 (24.4)	119 (33.1)	34 (9.4)	10 (2.8)	1.30 (32.50)
Malnutritional status in our city [Babol, Iran] and the increment of the risk of the gastric cancer due to it, caused me to have fear and anxiety	84 (23.3)	112 (31.1)	135 (37.5)	27 (7.5)	2 (0.6)	1.30 (32.50)
I feel extremely anxious when I think of an individual and has family problems after a possible risk of the incidence of gastric cancer	76 (21.1)	90 (25)	140 (38.9)	50 (13.9)	4 (1.1)	1.48 (37.00)
Heavy costs of the treatment of the probable gastric cancer made me and my family members feel anxious and fearful.	86 (24.2)	77 (21.4)	121 (33.6)	64 (17.8)	11 (3.1)	1.54 (38.50)
I get anxious and disappointed when I think about the surgery, chemotherapy and radiotherapy due to gastric cancer	67 (18.6)	73 (20.3)	115 (31.9)	95 (26.4)	10 (2.8)	1.74 (43.50)
Thinking about my family members' and my close relatives' stress about gastric cancer terrifies me	53 (14.7)	66 (18.3)	125(34.7)	101 (28.1)	15 (4.2)	1.88 (47.00)

Notice: The numbers in the last column of this table are expressed to mean (percentage) and the rest of the columns are expressed

to frequency (percentage).

The results of the statistical test ANOVA showed a significant difference between the mean score of perceived severity construct in terms of educational level, as the participants with college degree had higher perceived severity scores. Meanwhile, the women with both constructs of fear and perceived severity had higher mean score, but this difference was not statistically significant (table 2).

Pearson’s correlation coefficient showed that there was a significant inverse correlation between perceived severity with nutritional high risk behavior associated with gastric cancer in significant level of 0.05 (r=-0.165, P=0.027). Multivariable regression showed that by controlling the variables: age, sex, job and level of education as the predictor university level education (P=0.001, β=0.172) and sex (P=0.045, β=0.105) is a more significant predictor for perceived severity construct (table 3). In a study of predicting the nutritional high risk behaviors associated with gastric cancer by constructs of perceived severity and fear, linear regression test showed that together these constructs predicted 38% of the variance of above high risk behaviors.

**Table 2:** Evaluation of the relationship between fear and perceived severity with demographic variables on the nutritional high risk behavior on the risk of gastric cancer in patients over 30 years referred to urban health care centers, Babol, Iran

** Constructs** **Variable attributes**	**Fear** ** Mean±SD pvalue**		**Perceived severity** **Mean±SD pvalue**	
**Education level** Below high school High school University degree	9.41±4.95 8.94±4.77 9.46±4.33	0.623	16.61±3.03 16.10±3.10 17.32±2.68	*0.003*
**Sex** Male female	9.11±4.68 9.42±4.58	0.523	16.43±3.08 17.02±2.80	0.60
**Age (years)** 31-40 41-50 51-60 Over 60	9.28±4.51 9.43±4.80 9.05±5.21 8.60±4.35	0.943	16.89±2.75 16.20±3.54 16.87±2.98 15.90±2.76	0.273
**Job** Housekeeper Employee Self-employed Retired or jobless	9.44±4.50 10.00±4.22 8.86±4.96 9.26±4.63	0.350	17.09±2.81 16.70±2.65 16.48±3.20 16.30±2.31	0.356
**Father's level of education** Illiterate Below high school High school University degree	9.81±4.69 9.07±4.76 8.93±4.37 8.26±3.67	0.336	16.81±3.16 16.85±2.83 15.95±2.86 16.86±2.75	0.316
**Mother's level of education **Illiterate Below high school High school University degree	9.19±4.80 9.36±4.52 8.92±4.41 10.71±2.87	0.818	16.72±2.94 17.02±2.90 15.50±2.88 16.14±3.84	0.089

**Table 3:** Table of regression analysis predicting the perceived severity construct with underlying variables

** Model** **Predictor**	**Univariate** ** Model**			**Multivariate** ** Model**		
	**Standardized Coefficients** **Beta**	**Unstandardized Coefficients** **B**	**P-value**	**Standardized Coefficients** **Beta**	**Unstandardized Coefficients** **B**	**P-value**
Constant	-	15.378	< 0.001	-	15.374	< 0.001
**Sex** Man Women	- 0.103	- 0.611	- 0.47	- 0.105	- 0.618	- 0.045
**Level of Education** Below high school High school University degree	- -0.079 0.123	- -0.492 0.738	- 0.229 0.063	- - 0.172	- - 1.032	- - 0.001

## Discussion

In this study, the lowest level of fear in the field of gastric cancer was about fear of probability of developing gastric cancer caused by bad nutritional status in the surrounding area in the same way as several of the participants’ townmates. In addition, worrying about individual and family problems ranked the second lowest. In his study, Cherati showed that increase in eating salty foods and lack of fruits in diet are the two important causes of developing gastric cancer in Mazandaran Province, Iran, especially in the city of Babol (25). Baziar et al. found that the individual and the family problem of a patient with gastric cancer is the patient or his/her family members’ absence from work or even job dismissal; an issue that threatens the financial situation of patients’ family. Moreover, Baziar et al. believe that gastrointestinal cancers especially gastric cancer are among the most costly of all cancers in terms of treatment expenses (26). 

In Vrinton’s study, majority of the participants (59%) stated their agreement or in several cases strong agreement about the fact that they feared cancer more than other diseases. Furthermore, the cancer fear indicators were significantly higher in women (ORs between 1.15 and 1.48). (27) In the present study, as well, the mean score of fear in females showed to be higher than in males. Somi’s study in East Azerbaijan Province in Iran indicated that 72 percent of people suffering from gastric cancer had severe atrophy, while majority of them knew that atrophy was an important symptom of cancers (28). Accordingly, in the present study, the overall level of fear of developing cancer was very low in all of the six questions asked from the participants. This means that due to lack of sufficient awareness about gastric cancer, these people did not have the normal and reasonable stress and anxiety required to take preventive measures. In other words, there was not enough and effective fear to avoid high risk nutritional behaviors associated with gastric cancer among the participants.

Safarzadeh’s study on stress management in women with breast cancer showed that fear of death and fear of treatment and its side effects are of the major factors that affect the quality of life in these people (29). Scientists believe that most cancers can be prevented by changing nutritional behaviors. For instance, 90 percent of colorectal cancers can be prevented through diet changes (14). In this regard, in the present study, the lowest level of perceived severity in the field of probability of suffering from gastric cancer was acquired in reaction to the statement referring to existence of severe abdominal pain as a symptom of gastric cancer. The second lowest level belonged to the statement about the high prevalence of gastric cancer Babol, Iran. Kourneya and Hellsten noted that contrary to those who believed colon cancer was a very serious disease and as a result were more motivated to do exercises with a significant difference, those who did not believe in the severity of this disease were not motivated to do exercises based on their beliefs regarding the effectiveness or ineffectiveness of doing exercise in reducing the risk of colorectal cancer (30). This finding is consistent with protection motivation theory (PMT), which suggests that individuals who perceive a threat will be more motivated to change their behavior if they believe in the effectivity of the coping strategy offered to them for reducing that threat (31). In a study on breast cancer in Australia based on PMT, Ralf et al. concluded that perceived vulnerability, severity and response efficacy appeared to be the most influential factors on women’s decision to take or not to take selective estrogen reuptake modulators (SERMs) (32). Finally, Namdar’s study on cervix cancer based on health belief model showed that 66 percent of participants acquire good mean score for perceived severity (15.15 out of 20) (33). This is in conformity with the present study in which the findings indicate that the level of perceived severity in participants with university level education is higher than in participants with lower level of education (high school and below). That is to say, the mean score of perceived severity among participants with university degree was more than those with high school or lower level of education (17.32 out of 23), in as much as this education-based score causes a statistically significant difference. Namdar’s study on cervix cancer in Fasa, Iran (33) and Ruta Everatta et al.’s study (2012) in Lithuania on alcohol consumption as a risk factor for developing gastric cancer (34) also represents the same issue. In addition, the results of the present study indicate that level of fear in the age group of 41-50 years and perceived severity level in the age group of 31-40 years are higher than other age groups.

It appears that people in age group of 31-40 years are better aware of the seriousness of gastric cancer due to the high level of awareness they acquire in their residential area and work environment especially as a result of information and education given by healthcare centers and also because of their active presence in the society. However, since they are younger than other the elder age groups and accordingly have a longer life expectancy, their level of fear is almost lower. On the other hand, people in age group 41-50 years who became inactive in the society and with regard to the growth of the children and lack of necessary visit to healthcare centers, they receive less health education than others and consequently do not perceive the seriousness of the disease properly. Yet, because of advanced age and incidence of a number of symptoms and complications of some diseases, their level of fear for most chronic diseases especially cancers increases. Stimulation of lower level of fear in men is also reasonable (lower level of fear decreases an individual’s motivation for health protective behaviors). In the analysis of prediction rate of high risk nutritional behaviors associated with gastric cancer using fear and perceived severity constructs, the linear regression test showed that together these two constructs predict 38 percent of the aforementioned behaviors. This finding is in conformity with the results from Barr’s study on influenza pandemic in Australia (2008) in which fear construct predicted 45.5 percent of preventive behaviors (35). The results of the present study showed that there was a negative correlation between high risk nutritional behavior and perceived severity (P=0.005). This negative correlation indicates that as the individual’s belief in seriousness of the health threat increases, his/her high risk behaviors decrease accordingly.

The limitation of the present study is that it is conducted on individuals with 30 years of age and older. Thus, it is suggested that another study be held in this field with younger participants. The present study clearly showed that the participants’ level of fear and anxiety for the probability of gastric cancer resulted from bad nutritional status within their residential area in Babol, Iran was low (32.5%) and besides, their level of stress and anxiety for individual and family problems with developing gastric cancer was also low (about 37%).

This low level of fear and anxiety underlies the high risk nutritional behavior associated with gastric cancer and as a result increases the risk of developing this disease. On the other hand, the participants’ scores regarding their reactions toward the prevalence and seriousness of gastric cancer in Babol, Iran was almost low (62%) which indicates their lack of awareness about this disease and the people suffering from it in this city. Hence, the authors believe that serious measures should be taken for planning and developing preventive strategies for gastric cancer and informing people about all kinds of carcinogenic foods considered gastric cancer risk factors. Accordingly, improving awareness, attitude and performance of people of the society about unhealthy foods as well as healthy foods that prevent gastric cancer through national health policies such as holding public training courses especially for mothers who are responsible for managing the family food basket along with the use of mass media specifically the television to inform people is recommended. Considering the low level of fear and the relatively low level of perceived severity regarding gastric cancer and consequently lack of taking appropriate nutritional behaviors that prevent this disease, application of protection motivation theory to improve awareness and provide accurate information as well as the emphasis on the role of doctors and health experts of healthcare centers in educating the clients and encouraging them to take preventive behaviors especially in terms of nutrition is suggested. The perceived severity and fear that are two important constructs of protection motivation theory, have a significant role in taking high risk nutritional behaviors associated with gastric cancer. Increasing perceived severity and fear with the purpose of decreasing high risk nutritional behaviors associated with gastric cancer can be regarded as an important principle in planning educational programs to prevent this disease. 

The findings of this study showed the effective implementation of perceived severity as the important construct of protection motivation theory in predicting the behaviors. Consequently, this model can be used in developing educational programs and interventional techniques in urban and rural healthcare centers as well as schools, companies and offices to change knowledge, attitude and performance in preventing gastric cancer with the participation of clients and related experts.

## Notes

**Funding:** This article was extracted from the PhD. dissertation of Seyed Jalil Seyedi-Andi on health promotion and education presented at the Faculty of Health, Shahid Sadoughi University of Medical Sciences and Health Services, Yazd.

## References

[1] Niknam M, Azadbakht L (2012). Nutrition and gastric cancer: a review of epidemiologic evidences. J Health Syst Res.

[2] Najimi A, Moazemi Goudarzi A (2012). Healthy lifestyle of the elderly: a cross-sectional study. J Health Syst Res.

[3] Matsushita T, Matsushima E, Maruyama M (2005). Psychological state, quality of life, and coping style in patients with digestive cancer. Gen Hosp Psychiatry.

[4] Brenner H, Rothenbacher D, Arndt V (2009). Epidemiology of stomach cancer. Methods Mol Biol.

[5] Yang L (2006). Incidence and mortality of gastric cancer in China. World J Gastroenterol.

[6] Sadat Asmarian N, Kavousi A, Salehi M, Mahaki B (2012). Mapping of Stomach Cancer rate in Iran using Area-to-Area Poisson Kriging. J Health Syst Res.

[7] Ghadimi R, Taheri H, Suzuki S (2007). Host and environmental factors for gastric cancer in Babol, the Caspian Sea coast, Iran. Eur J Cancer Prev.

[8] Tokudome S, Hosono A, Suzuki S (2006). Helicobacter pylori infection as an essential factor for stomach cancer. Asian Pac J Cancer Prev.

[9] Tokudome S, Ghadimi R, Suzuki S (2006). Helicobacter pylori infection appears the prime risk factor for stomach cancer. Int J Cancer.

[10] Sanei MH, Sanei B, Mahzoni P, Chahreai A (2007). Comparison of histopathologic findings of non-tumoral gastric mucus of patients with gastric cancer and patients with chronic gastritis. J Shahrekord Univ Med Sci.

[11] Bingam S (2005). What do people eat? Adventures in nutritional epidemiology. Nutr Bull.

[12] Tsugane S (2005). Salt, salted food intake and risk of gastric cancer: epidemiologic evidence. Cancer Sci.

[13] Tsugane S, Sasazuki S (2007). Diet and the risk of gastric cancer: review of epidemiological evidence. Gastric Cancer.

[14] Mirbazegh SF, Rahnavard Z, Rajabi F (2012). The effect of education on dietary behaviors to prevent cancer in mothers. J Res Health.

[15] Briggs M, Safaii S, Beall DL (2003). Position of the American dietetic association, society for nutrition education, and American school food service association--nutrition services: an essential component of comprehensive school health programs. J Am Diet Assoc.

[16] Lynch L, Happel B (2008). Implementation of clinical supervision in action: part 2: Implementation and beyond. J Ment Health Nurs.

[17] Agha Moulaei T, Eftekhar H, Mohammad K (2005). Application of health belief model to behavior change of diabetic patients. Payesh.

[18] Milne S, Sheeran P, Orball SH (2000). Prediction and intervention in health-related behavior: a meta-analytic review of protection motivation theory. J Appl Soc Psychol.

[19] Sharifirad GH, Yarmohammadi P, Morowati Sharifabad MA, Rahayi Z (2011). The Status of preventive behaviors regarding influenza (A) H1N1 pandemic based on protection motivation theory among female high school students in Isfahan, Iran. J Health Syst Res.

[20] Floyd DL, Prentice Dunn S, Rogers RW (2000). A meta-analysis of research on protection motivation theory. J Appl Soc Psychol.

[21] MacDonell K, Chen X, Yan Y (2013). A protection motivation theory-based scale for tobacco research among Chinese youth. J Addict Res Ther.

[22] Baghianimoghadam MH, Mohammadi S, Mazloomi Mahmoudabad S, Norbala MT (2011). The Effect of education based on protection–motivation theory on skin cancer preventive practices among female high school students in Yazd. J Horizon Med Sci.

[23] Baghianimoghaddam MH, Mohammadi S, Norbala MT, Mazloomi S (2010). The study of factors relevant to skin cancer preventive behavior in female high school students in Yazd based on protection motivation theory. Knowledge Health.

[24] Brian T, McClendon, Prentice-Dunn S (2001). Reducing skin cancer risk: an intervention based on protection motivation theory. J H Psych.

[25] Yazdani Charati J, Zare S, Ghorbanpour E, Shabankhani B (2009). Demographic and geographical pattern of mortality rate from stomach cancer and related factors in Mazandaran province from 2001 to 2005. J Mazandaran Univ Med Sci.

[26] Bazyar M, Barfar E (2013). Cost analysis for cancer subgroups in Kerman. Iran J Epidemiol.

[27] Vrinten C, van Jaarsveld CH, Waller J, von Wagner C, Wardle J (2014). The structure and demographic correlates of cancer fear. BMC Cancer.

[28] Somi MH, Alizadeh N, Farhang S (2010). Diagnostic and treatment process of gastric cancer and setbacks in East Azerbaijan (2006). J Tabriz Univ Med Sci.

[29] Safarzade A, Roshan R, Shams J (2012). Effectiveness of stress management and relaxation training in reducing the negative affect and in improving the life quality of women with breast cancer. Res Psychol Health.

[30] Courneya KS, Hellsten LAM (2001). Cancer prevention as a source of exercise motivation: an experimental test using protection motivation theory. Psychol Health Med.

[31] Rogers RW, Prentice-Dunn S, Gochman DS (1997). Protection motivation theory. Handbook of health behavior research I: Personal and social determinants.

[32] Ralph AF, Ager B, Bell ML (2014). Women's preferences for selective estrogen reuptake modulators: an investigation using protection motivation theory. Patient Educ Couns.

[33] Namdar A, Bigizadeh S, Naghizadeh MM (2012). Measuring health belief model components in adopting preventive behaviors towards cervical cancer. J Fasa Univ Med Sci.

[34] Everatt R, Tamosiunas A, Kuzmickiene I (2012). Alcohol consumption and risk of gastric cancer: a cohort study of men in Kaunas, Lithuania, with up to 30 years follow-up. BMC Cancer.

[35] Barr M, Raphael B, Taylor M (2008). Pandemic influenza in Australia: using telephone surveys to measure perceptions of threat and willingness to comply. BMC Infect Dis.

